# Standardized Patient Surgical Marking Session: An Under-Utilized Plastic Surgery Resident Educational Tool

**DOI:** 10.1093/asjof/ojad083

**Published:** 2023-09-14

**Authors:** Ellen C Shaffrey, Samuel O Poore

In 2022, Bigarella et al highlighted in an article entitled “The Impact of Obesity on Plastic Surgery Outcomes: A Systematic Review and Meta-analysis” that obesity leads to more complications and a greater incidence of revision operations.^1^ Unfortunately, the incidence of obese patients is dramatically increasing in the United States, with over two-thirds of the population being overweight or obese.^2^ As a result, more patients will present to plastic surgeons for body contouring operations.

Aesthetic surgery, including body contouring procedures (BCPs), is noted to have the greatest opportunity for resident educational growth, given that plastic surgery residents, particularly those without resident aesthetic clinics, leave training with the least amount of direct participation in aesthetic operations.^3^ As a result, residents note lower levels of surgical confidence.^4^ Specific to BCPs, Malyar et al demonstrated low confidence in performing BCPs, which persists among early career plastic surgeons after graduation.^5^

Surgical markings are potentially the most critical aspect of an aesthetic operation as they determine the surgical plan and reflect special considerations for each patient, particularly in obese patients, whose risk of postoperative complications is higher than the general population. In general, residents have few opportunities to independently perform surgical markings during training, as not all programs have a resident-led aesthetic clinic. One underutilized educational resource that has yet to be implemented in plastic surgery is the use of standardized patients (SPs). Using SPs for medical education is one of the leading methods of training future doctors in diagnostic and examination skills. Over 75% of medical schools report using SPs in their medical curriculum as they provide a range of experiences for learners, including history taking, counseling, and physical examination techniques.^1^ Within residency, SPs have also been used to evaluate clinical and interpersonal skills, such as competency in obtaining informed consent.^2^ However, SPs have yet to be utilized to educate plastic surgery residents. To overcome this gap in plastic surgery training, here we describe utilizing SPs for an educational marking activity for 5 common BCPs.

Residents at a single institution performed an SP marking activity over the course of 1 afternoon for the following BCPs: breast reduction, mastopexy, brachioplasty, abdominoplasty, and thighplasty. Five SPs were recruited for each marking encounter after meeting specific criteria (ie, female SPs ranging between 30 and 60 years old with a BMI >25 and who were comfortable with varying degrees of exposure). Residents were divided between 5 rooms and rotated through each marking experience. Only 1 resident performed the markings, using a chalk-based marking pen, to avoid traumatizing the skin. Residents were expected to treat the encounter as if they were faculty marking in the preoperative area. A faculty member was present in each room and observed the resident's markings and technique to provide feedback after the resident completed the encounter. The SP delivered feedback on professionalism and communication.

After completing the activity, residents completed survey-based feedback regarding its educational utility. Feedback consisted of confirming the marking steps for each procedure, discussing how to best handle tissue while marking, and important points to bring up to patients, such as baseline asymmetries and locations of scars. All residents felt that the exercise would improve a resident's ability to perform markings in their future careers and would recommend it be incorporated into the plastic surgery curriculum. Comments on what residents felt were most helpful included getting feedback from faculty regarding markings right away, being able to ask questions without compromising patient confidence, independently performing the markings, and collecting tips and pearls from faculty. The majority of the residents felt that working with SPs was very realistic and should be continued at least yearly.

In this letter, we describe the first SP educational experience in plastic surgery, where residents independently performed markings for BCPs and received faculty and SP feedback. Continued implementation of SPs in plastic surgery training is needed to maximize resident education.
